# Cardiac Mechanics in Isolated Bicuspid Aortic Valve Disease With Normal Ejection Fraction: A Study of Various Valvular Lesion Types

**DOI:** 10.1097/MD.0000000000002085

**Published:** 2015-10-30

**Authors:** Xijun Zhang, Meihua Zhu, Tao He, Jianjun Yuan, Haohui Zhu, Dennis E. Morrisroe, Muhammad Ashraf, David J. Sahn

**Affiliations:** From the Department of Ultrasonography (XZ, JY, HZ,) The People's Hospital of Zhengzhou University, Zhengzhou, Henan, China; Pediatric Cardiology (MZ, MA, DJS), Oregon Health & Science University, Portland, Oregon; Department of Medicine I and Clinical Chemistry (TH), University of Heidelberg, Heidelberg, Germany; Hudson's Bay Medical Group (DEM), Vancouver, Washington.

## Abstract

Aortic stenosis (AS) and aortic regurgitation (AR) are associated with congenital isolated bicuspid aortic valve (BAV) disease. The chronic pressure overload of AS and the volume overload of AR are known to impair the left ventricular function. This study assessed whether two-dimensional speckle tracking echocardiography (2D-STE) is capable of detecting the myocardial dysfunction associated with BAV caused by various aortic valve lesions in patients retaining normal ejection fraction (EF).

Thirty-two isolated BAV patients and 20 healthy tricuspid aortic valve (TAV) volunteers were recruited. BAV patients were divided into 4 subgroups based on aortic valvular lesion types: normal function (NF) group, isolated AS group, isolated AR group, and a group who had both AS&AR. Myocardial strain and degree of twist were analyzed and compared between the BAV and TAV groups, as well as between valvular lesion groups and the NF group.

Compared with healthy TAV controls, global radial strain (GRS), global circumferential strain (GCS), global longitudinal strain (GLS), and twist angle absolute values were lower in the BAV group (*P* < 0.05). The AS, AR, and AS&AR groups all demonstrated a significant decrease in GRS and GCS when compared with the TAV group. The AS and AS&AR groups demonstrated lower GLS than the TAV group, and the smallest degree of twist was detected in the AR group. There were no significant differences between the NF and TAV groups. The AR and AS&AR groups demonstrated significant differences in multiple parameters of cardiac mechanics compared with the NF group.

2D-STE is able to detect altered cardiac mechanics associated with aortic lesion types in BAV patients with normal EF compared with normal TAV controls, and so can provide valuable information for clinical decision-making.

## INTRODUCTION

Bicuspid aortic valve (BAV) is the most common congenital cardiac malformation in adults.^1–3^ BAV is often associated with other forms of congenital cardiac diseases, including patent ductus arteriosus (PDA), ventricular septal defect, and aortic coarctation.^3–6^ Isolated BAV is defined as BAV without other congenital cardiac malformations. BAV is an ongoing pathological process; it has a high incidence of valvular lesions and aortopathy during the disease progress.^1,3,6^ The common valvular lesions of BAV are aortic stenosis (AS) and aortic regurgitation (AR). The chronic pressure overload of AS and the volume overload of AR are known to impair the left ventricular (LV) systolic and diastolic function, which can result in heart failure and death.^6–9^ The longitudinal evaluation of LV function in BAV patients is essential for patient management and intervention.^6,9–11^

Echocardiography is the primary imaging method used to monitor the cardiac dysfunction caused by valvular lesions of BAV. Traditionally, the clinical management and intervention decision-making regarding BAV lesions has mostly relied on the LV ejection fraction (LVEF). However, the LVEF is not reliable in patients with abnormal hemodynamic, LV hypertrophy, or ventricular dilatation.^7,12,13^

Many echocardiographic parameters of cardiovascular function have been used to explore the subtle changes of cardiac dysfunctions in valvular lesions, such as strain and twist. Tissue Doppler imaging (TDI) allows quantification of myocardial tissue velocities, from which strain can be obtained.^13^ However, TDI is limited because of the Doppler angle dependency. Speckle tracking echocardiography (STE) based on tracing acoustic markers within the myocardium on standard two-dimensional (2D) images provides strain and rotation parameters, which overcomes the limitation of TDI and has proven to be valuable in evaluating cardiac function.^14–16^ Investigations of AS and AR in tricuspid aortic valve (TAV) patients revealed abnormal strain and rotation values when compared with normal healthy controls.^8,13^ Analysis of strain provides a powerful means of unmasking subtle myocardial dysfunction that is not detected by LVEF in early stages.^17–20^ However, the various acquired pathological causes of TAV AS/AR may have different effects on LV dysfunction compared with congenital BAV lesions, so the findings of TAV deformation abnormalities cannot be reliably applied to BAV patients. Therefore, the aim of this study is to reveal the subtle changes of cardiac mechanics in various valvular lesion conditions of BAV in patients with normal EF.

## METHODS

### Subjects

This study was approved by our institutional review board and was conducted in compliance with institutional human research policy. All subjects were given written informed consent before image acquisitions. From August 2012 to August 2014, 52 subjects were recruited in this study, including 32 isolated BAV patients (25 male, 7 female, age 46 ± 14 years), and 20 age- and sex-matched healthy TAV volunteers (14 male, 6 female, age 39 ± 9 years) (Table 1). Exclusion criteria were as follows: diagnosed hypertension, diabetes, cardiomyopathy, hypercholesterolemia, coronary artery disease, and/or other congenital cardiovascular conditions. All subjects included in both the groups were in sinus rhythm. The EF of the healthy controls and the BAV patients was determined by Simpson's biplane method (all subjects’ EF >50%).

**TABLE 1:**
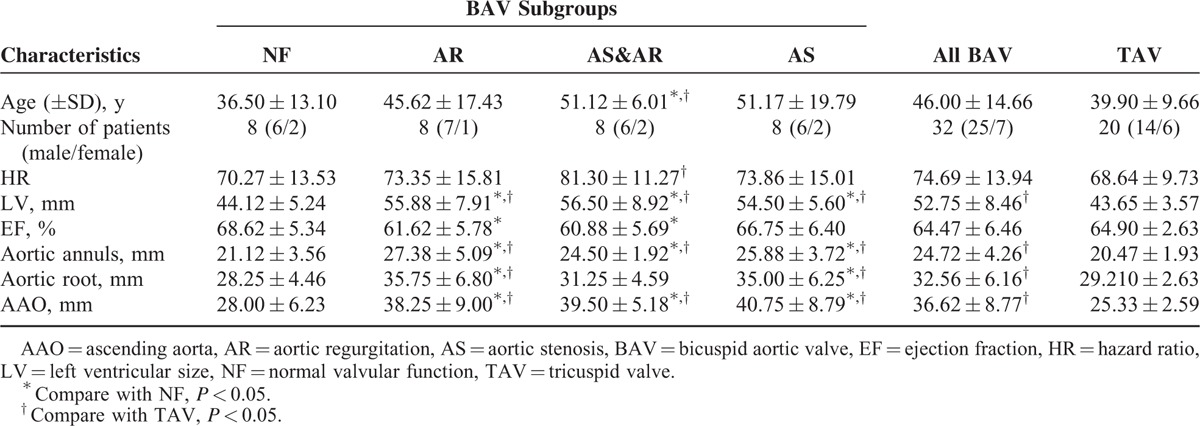
Demographic and Basic Echocardiographic Data of BAV Patients and TAV Controls

All subjects underwent a standard echocardiographic evaluation to exclude other heart disease and to define the valvular lesion of BAV. AS and AR were defined according to American Society of Echocardiography (ASE) guidelines.^21,22^ All 32 patients with isolated BAV were equally divided into 4 subgroups based on the valvular lesion: normal function BAV (NF group), AS group, AR group, and AS&AR group (Table 1).

### Image Acquisition and Analysis

All patients underwent complete 2D and Doppler echocardiographic examinations according to ASE recommendations,^23^ using a Vivid 7 Digital Ultrasound System (GE Medical Systems, Horten, Norway). Aortic valve morphology was evaluated in the parasternal long and short-axis views. Upon the exclusion of other congenital heart disease, isolated BAV was diagnosed when only 2 cusps were clearly identified in systole and diastole in the short-axis view. Patients with severe aortic dystrophic calcification, fusion of the commissures, or any other valve abnormality attributable to rheumatic disease were excluded from the study. Aortic size was assessed at 3 levels (annulus, Valsalva sinuses, and ascending aorta at 3 cm from the valve).

**FIGURE 1:**
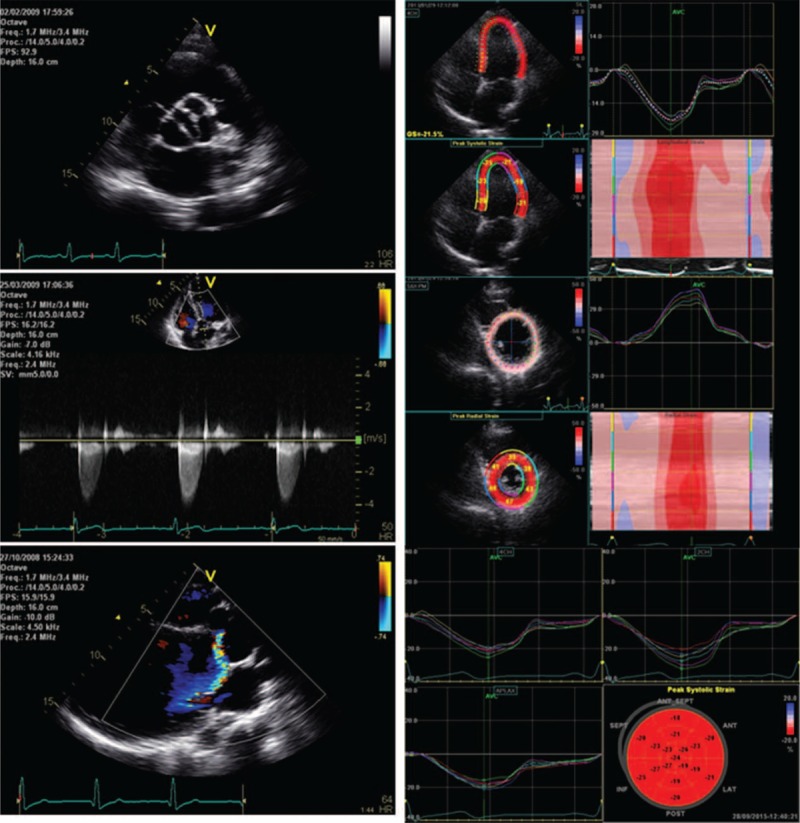
Left: bicuspid aortic valve, aortic stenosis, and aortic regurgitation; right: specking tracking on 4-chamber view, tacking on short-axis papillary muscle level, and the average of peak strain.

The presence of AR was assessed on Color Doppler by the use of standard criteria^24^ and graded as mild, moderate, or severe, according to the regurgitation jet size. Aortic valve peak velocity was assessed by continuous wave Doppler in multiple views. AS severity, graded using current guidelines,^23^ was classified into mild, moderate, or severe (Fig. 1). NF BAV valve was defined as having an aortic jet velocity ≤2.5 m/s and no AR signal detected by Color Doppler at multiple views.

LV dimensions were measured in the parasternal long-axis view. LVEF was calculated by Simpson's biplane method at apical views with normal LVEF defined as >50%.

Speckle-tracking echocardiography was performed to assess myocardial strain during systole. Two-dimensional recordings were collected with frame rates ranging from 50 to 80 frames/s during a brief breath hold. Apical long-axis view, and apical 4-chamber and 2-chamber views were recorded for longitudinal strain analysis. Parasternal short-axis views at mitral valve, papillary muscle, and apical level were acquired for circumferential strain, radial strain, and twist analysis. Three consecutive cardiac cycles were recorded as 2D cine loops and were acquired as raw data format. LV mechanics including strain and rotation were analyzed by a single observer blinded to the clinical findings and the valvular lesions. Off-line analyses of myocardial mechanics from archived image loops with the highest frame rate were performed using commercially available analysis software (EchoPAC; GE Medical Systems). The region of interest (ROI) of the LV was defined by tracking the endocardial and epicardial borders. The ROI width was adjusted as needed to fit to the wall thickness, as previously described.^25–27^ The tracking quality of each segment was indicated by the software, and segments with insufficient tracking quality were excluded. Peak strain values at 17 segments and global strain were acquired. Global longitudinal strain (GLS) was calculated from the average of GLS at 3 apical long-axis views. Global circumferential strain (GCS) and global radial strain (GRS) were calculated from the average of GCS and GRS at 3 short-axis view levels. Twist was calculated from the difference of rotation at apical and basal short-axis views (Fig. 1).

### Statistical Analysis

Data are expressed as mean ± standard deviation. All data obtained from the BAV group were compared with that obtained from the TAV group using the independent samples test (SPSS 13.0; SPSS Inc, Chicago, IL). Comparisons between the subgroups, along with comparisons between subgroups and the TAV group, were carried out by the bootstrapping method from the R language (R Foundation for Statistical Computing, Vienna, Austria).^28,29^ All the data were graphed in Microsoft Excel (Microsoft Corporation, Redmond, WA). Significance levels were defined at *P* *<* 0.05.

Intraobserver analysis was conducted 2 months after completion of the initial measurements. For interobserver variability, a second observer analyzed 20% of the images. Intraobserver variability and interobserver variability were assessed using the intraclass correlation coefficient (ICC).

## RESULTS

### Basic Demographic and Echocardiographic Data

There were no differences in age, sex, heart rate, and EF between the BAV group as a whole and the TAV group (Table 1). The LV sizes of the BAV group patients (52.75 ± 8.46 mm) were significantly larger than those of the TAV group (43.65 ± 3.57 mm) (*P* < 0.05). The aortic annulus, the aortic sinus, and the ascending aorta were significantly dilated in BAV groups when compared with the TAV group (all *P* < 0.05) (Table 1).

When compared with the TAV group, there was no significant decrease in strain and twist in the NF group. However, the AS&AR group included older patients (51 ± 6 years) with higher heart rates (81 ± 11) when compared with the TAV group (age: 36 ± 13 years, hazard ratio: 68 ± 9). The LV sizes and the aortic diameters in the AR group, AS&AR group, and the AS group were larger than those of the TAV group (Table 1).

When compared with the NF group, the patients’ ages in the AS&AR group were older (Table 1). Even the EF in the valvular lesion groups (AS, AS&AR, and AR) was slightly lower than that of the NF group, but they were comparable with the TAV group and within a normal range. The LV sizes and the aorta diameters at different levels in the valvular lesion groups were bigger than that of the NF group (Table 1).

### Cardiac Mechanics of BAV Versus TAV

**FIGURE 2:**
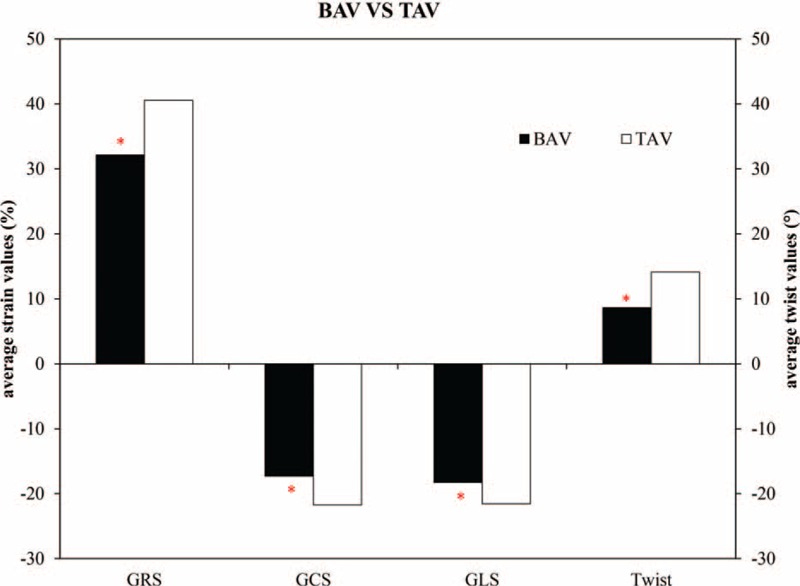
Comparison of strain values and twist values BAV group and TAV group. BAV = bicuspid aortic valve, GCS = global circumferential strain, GLS = global longitudinal strain, GRS = global radial strain, TAV = tricuspid aortic valve. ^∗^*P* < 0.05.

Compared with healthy TAV controls, GRS, GCS, and GLS had lower absolute values in the BAV group (*P* < 0.05; Table 2). Twist angle was smaller in BAV group (9.12° ± 3.98°) than that of the TAV group (14.12° ± 5.46°) (*P* < 0.05; Table 2) (Fig. 2).

**TABLE 2:**
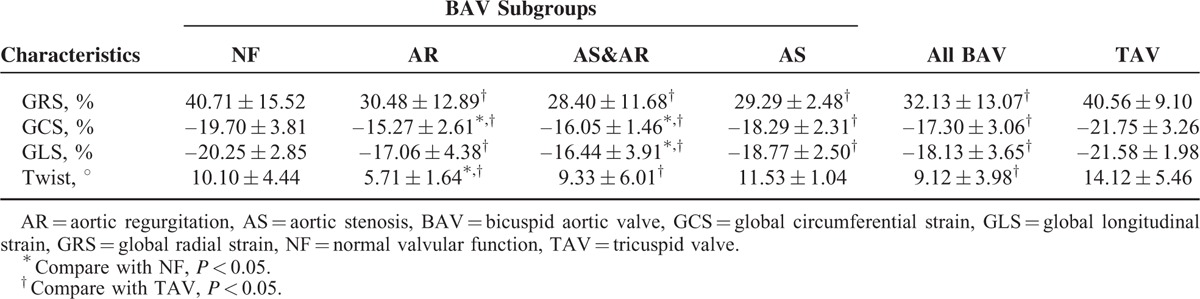
Global Strain Values and Twist Values of BAV Patients and TAV Controls

**FIGURE 3:**
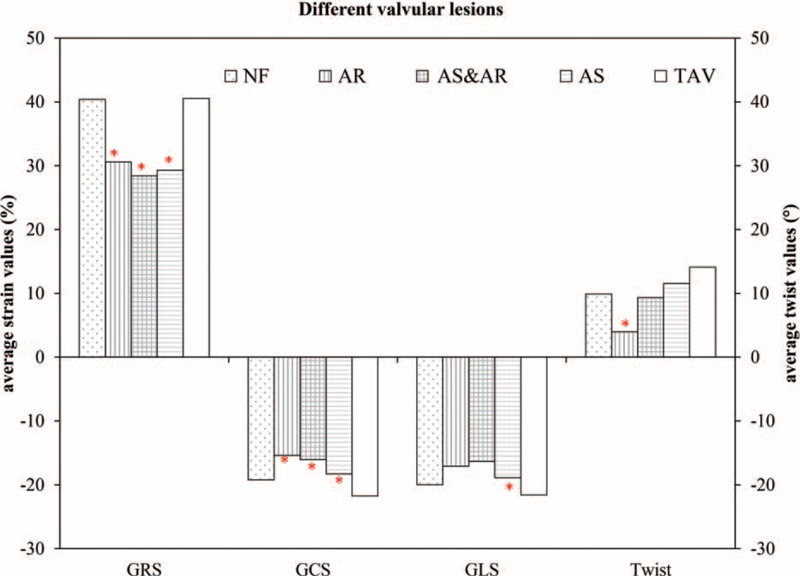
Comparison of strain values and twist values in different BAV valvular lesion groups and (TAV group. BAV = bicuspid aortic valve, GCS = global circumferential strain, GLS = global longitudinal strain, GRS = global radial strain, TAV = tricuspid aortic valve. ^∗^*P* < 0.05.

In the subgroups, when compared with the TAV group, the AS, AR, and AS&AR groups all demonstrated significantly decreased GRS and GCS. The AS and AS&AR groups also exhibited significantly lower GLS than the TAV group, but the other lesion groups did not. The AR group had the smallest degree of twist among all the groups and was significantly lower than that of the TAV group; the AS&AR group also demonstrated lower twist when compared with the TAV group (Table 2, Fig. 3).

The strain values and twist in the NF group were similar to those in the TAV group; however, there were significant differences between the NF and other BAV subgroups. When compared with the NF group, the AR group showed significantly lower values in terms of GCS (*P* = 0.017) and twist (*P* = 0.028), and the AS&AR group showed significantly lower values in terms of GCS (*P* = 0.019) and GLS (*P* = 0.043) (Table 2).

### Interobserver Variability and Intraobserver Reproducibility

**FIGURE 4:**
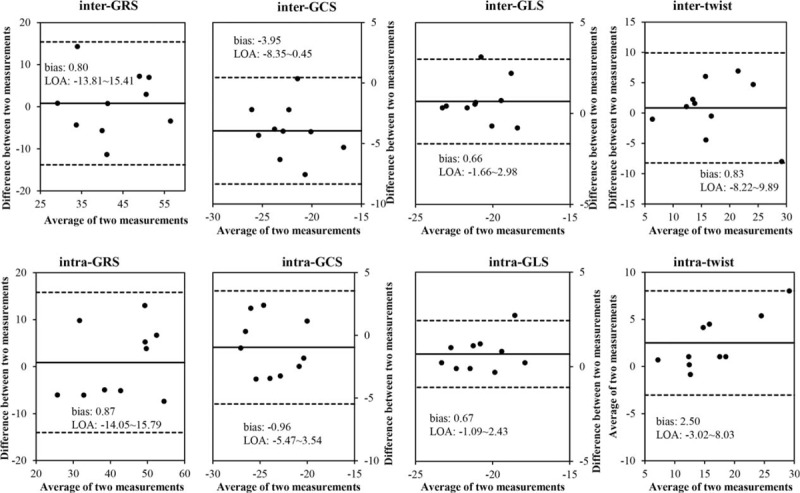
Inter- and intraobserver analyses for GRS, GCS, GLS, and twist. GCS = global circumferential strain, GLS = global longitudinal strain, GRS = global radial strain.

Excellent correlations were found between initial measurements and values generated from the same observer 2 months later, with high *R* values for GRS, GCS, GLS, and twist (Table 3; *P* < 0.05). ICC between initial values and reanalyzed values demonstrated good reproducibility of strain analysis. Interobserver analyses demonstrated excellent correlations between the 2 observers’ measurements. ICC between the 2 measurements also demonstrated good agreement (Table 3). Bland-Altman plots revealed minimal variation between measurements for both inter- and intraobserver analyses (Fig. 4). Measurements of GLS and twist showed relatively better reproducibility and smaller variability.

**TABLE 3:**
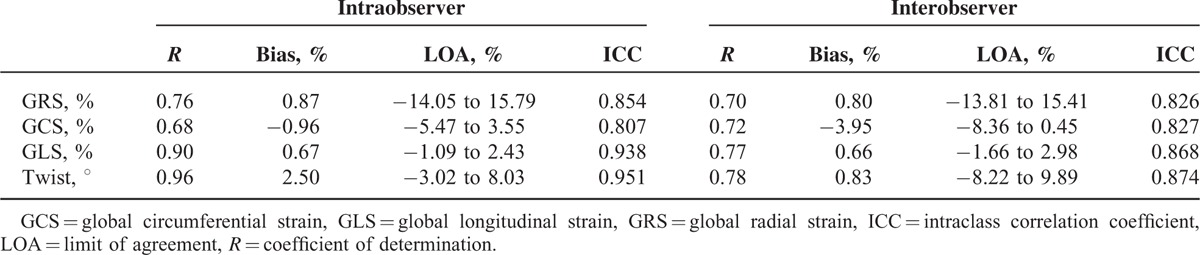
Inter- and Intraobserver Analyses for Global Strain and Twist Values

## DISCUSSION

BAV is an ongoing pathological process. Its valvular lesions, AS and regurgitation, and the anomalies of the aorta alter LV mechanics. During the natural course of BAV, the progressive rise in the volume and pressure load leads to impaired LV function. STE provides deformation information of the LV, which has been reported to be valuable in identifying preclinical or subtle myocardial dysfunction.^14–16,26,30^ STE has been used in TAV to evaluate cardiac mechanics in AS and AR patients, which revealed lower deformation in asymptomatic/symptomatic patients with normal LVEF when compared with healthy controls.^17–20^ Few reports are available on BAV cardiac mechanics,^31–33^ and none of them reported the subtle mechanical changes based on valvular lesion types.

In this study, the BAV group showed decreased radial, circumferential, and longitudinal strain, as well as twist when compared with the TAV group (*P* < 0.05). The aortic valve lesion groups contributed to the degeneration of LV mechanics (*P* < 0.05), whereas the normal valvular function (NF) BAV group showed no significant decrease in strain values and twist when compared with the TAV group. Santarpia et al^31^ showed that longitudinal, circumferential, and radial strain values were significantly lower in BAV, but found no statistical difference in torsion. The difference in findings between their study and our current study is due to the definition of normal valvular function of the recruited population. In our study, the NF group was defined as having aortic valve peak velocity <2.5 m/s and no regurgitation detected by Color Doppler in multiple views. Thus, our definitions of mild AS and AR were categorized as normal in the Santarpia study. Our more conservative definition of NF suggests that valvular lesions contribute to the dysfunction of LV as demonstrated by decreased strain values.

Most TAV valvular lesion studies have focused on GLS, which was proposed as the most sensitive and representative value of myocardial deformation.^17–20^ Kurt et al^33^ reported decreased GLS in BAV patients without valvular lesions and free of aortic distension (19.6 ± 1.1 vs 17.7 ± 0.9, *P* < 0.001). They proposed that BAV is not only a valvular disease, but also a ventricular disease. The current study demonstrated a lowered GLS in the NF group when compared with normal TAV (−20.25 ± 2.85 vs −21.58 ± 1.98); however, this did not reach statistical significance. With a larger sample size and smaller measurement variation, a significant decrease of GLS may have been revealed in our current NF group as well. The ventricular deterioration of BAV should not be overlooked. Circumferential strain and radial strain are both important parameters for cardiac deformation, as they represent different myocardial layers compared with LS, and they are proposed to be a sign of cardiac dysfunction at a later, more advanced state.^14^ The current study proposes that circumferential strain and radial strain are also important deformation parameters in BAV. Because BAV is a congenital heart disease, the chronic volume and pressure load or the deterioration of myocardium may result in decreased circumferential strain and radial strain. This was reflected in our study; GCS and GRS in the AR, AS, and AS&AR groups were significantly lower compared with the TAV group. In addition, inter- and intraobserver analyses revealed relatively better reproducibility and smaller variability in GLS and twist. This indicates that GLS and twist may be useful parameters of BAV deformation and warrants further exploration in multicenter longitudinal studies.

A previous study by Tzemos et al^32^ discovered that LV twist was significantly increased in the antepartum period of pregnancy in women with congenital BAV AS, except for those who experienced functional deterioration. The AS group in the current study showed no significant difference in twist when compared with normal TAV, but a relatively higher, although not significant, twist than the NF BAV group. However, the patients’ ages and sexes were different in the current study from Tzemos et al's. Because BAV is congenital, the progressive deterioration may result in increasing cardiac dysfunction with age. Further longitudinal investigation on cardiac mechanics in BAV is necessary. Previous aortic valvular studies have mainly focused on AS in TAV and BAV^32,34,35^; however, the current study finds that the AR group has the most significantly lowered twist, therefore monitoring of cardiac function related to AR is very important in BAV patients.

## LIMITATIONS

In addition to the possible limitations discussed above, the small number of patients and the single-center, cross-sectional nature of this study is the major limitation. Large multicenter, longitudinal studies with larger sample sizes in each subgroup will be necessary to definitively characterize the LV dysfunction in patients with BAV.

In addition, the absence of coronary artery disease of the recruited subjects in this study was based on patients’ medical history rather than on coronary angiography, which might limit the interpretation of the results. Differences in baseline clinical, demographic, and echocardiographic data could have potentially confounded the results in this current study. The severity of AS and AR in the BAV patients was not classified in the current study, which might present different effects on strain values due to the progressive nature of the diseases. 2D-STE has advantages over traditional LVEF and TDI-based techniques; however, its reliance on geometric assumptions limits its accuracy on tracking the myocardial motion. 3D-STE with higher temporal resolution would provide superior evaluation of subtle cardiac mechanic dysfunction.^14,16,30^ Future 3D-STE studies on BAV deformation are necessary.

## PERSPECTIVES

Early detection of myocardial dysfunction in patients with BAV may be useful for directing patients’ care and clinical decision-making. Earlier detection of subclinical myocardial dysfunction allows for identification of patients at risk of irreversible myocardial damage. Because a delay of surgery until symptoms develop was reported to be associated with postoperative risks of LV dysfunction and death,^20,36–38^ early detection of subclinical LV dysfunction may help clinicians choose optimal timing of intervention. Speckle-tracking echocardiography could pick up the early signs of myocardial dysfunction through analyses of strain, which should then prompt increased vigilance, including more frequent follow up in patients. The results of the current study indicate that maintaining normal valvular function is essential to prevent the progress of myocardial deterioration; once the AS or AR develops, the progressive deterioration of cardiac function is inevitable. Earlier pharmacological therapy and surgical procedures to prevent the degeneration of valvular lesions and LV remodeling are important. 2D-STE may be a future recommendation for BAV patient follow-up because it could allow for earlier detection of myocardial dysfunction. 2D-STE would ensure that more BAV patients receive the recommended surgical interventions before the occurrence of irreversible myocardial damage.

## CONCLUSIONS

2D-STE was able to detect different cardiac mechanics associated with aortic lesion types in BAV patients with normal ejection fraction, and stands to provide valuable information for clinical decision-making.

## References

[1] Tzemos N Therrien J Yip J Outcomes in adults with bicuspid aortic valves. *JAMA* 2008; 300:1317–1325. 18799444 10.1001/jama.300.11.1317

[2] Roberts WC The congenitally bicuspid aortic valve. A study of 85 autopsy cases. *Am J Cardiol* 1970; 26:72–83. 5427836 10.1016/0002-9149(70)90761-7

[3] Braverman AC Guven H Beardslee MA The bicuspid aortic valve. *Curr Probl Cardiol* 2005; 30:470–522. 16129122 10.1016/j.cpcardiol.2005.06.002

[4] Agarwal A Khandheria BK Paterick TE Left ventricular noncompaction in patients with bicuspid aortic valve. *J Am Soc Echocardiogr* 2013; 26:1306–1313. 24044978 10.1016/j.echo.2013.08.003

[5] Cripe L Andelfinger G Martin LJ Bicuspid aortic valve is heritable. *J Am Coll Cardiol* 2004; 44:138–143. 15234422 10.1016/j.jacc.2004.03.050

[6] Zhu M Deng Y Liu Y Evaluation of aortic valve function in patients with bicuspid aortic valve with echocardiography. *Chin J Ultrasonogr* 2009; 18:748–750.

[7] Donal E Thebault C O’Connor K Impact of aortic stenosis on longitudinal myocardial deformation during exercise. *Eur J Echocardiogr* 2011; 12:235–241. 21245060 10.1093/ejechocard/jeq187

[8] Olsen NT Sogaard P Larsson HB Speckle tracking echocardiography for predicting outcome in chronic aortic regurgitation during conservative management and after surgery. *JACC Cardiovasc Imaging* 2011; 4:223–230. 21414568 10.1016/j.jcmg.2010.11.016

[9] Stewart WJ King ME Gillam LD Prevalence of aortic valve prolapse with bicuspid aortic valve and its relation to aortic regurgitation: a cross sectional echocardiografic study. *Am J Cardiol* 1984; 54:1277–1282. 6507297 10.1016/s0002-9149(84)80080-6

[10] Fenoglio JJ Jr McAllister HA Jr De Castro CM Congenital bicuspid aortic valve after age 20. *Am J Cardiol* 1977; 39:164–169. 835475 10.1016/s0002-9149(77)80186-0

[11] Sadee AS Beker AE Verheul HA Aortic valve regurgitation and the congenitally bicuspid aortic valve: a clinico-pathologic correlation. *Br Heart J* 1992; 67:439–441. 1622690 10.1136/hrt.67.6.439 PMC1024882

[12] McGowan JH Cleland JG Reliability of reporting left ventricular systolic function by echocardiography: a systematic review of 3 methods. *Am Heart J* 2003; 146:388–397. 12947354 10.1016/S0002-8703(03)00248-5

[13] Wang B Chen H Shu X Emerging role of echocardiographic strain/strain rate imaging and twist in systolic function evaluation andoperative procedure in patients with aortic stenosis. *Interact Cardiovasc Thorac Surg* 2013; 17:384–391. 23644729 10.1093/icvts/ivt171 PMC3715177

[14] Zhu M Streiff C Panosian J Regional strain determination and myocardial infarction detection by three-dimensional echocardiography with varied temporal resolution. *Echocardiography* 2015; 32:339–348. 24815184 10.1111/echo.12632

[15] Cyprien MM Deng Y Bi X The evaluation of diastolic function in early myocardial ischemia by strain rate imaging combined with high-dose dobutamine stress echocardiography. *Chinese J Ultrasound Med* 2011; 27:624–627.

[16] Streiff C Zhu M Panosian J Comprehensive evaluation of cardiac function and detection of myocardial infarction based on a semi-automated analysis using full-volume real time three-dimensional echocardiography. *Echocardiography* 2015; 32:332–338. 24930502 10.1111/echo.12643

[17] Lafitte S Perlant M Reant P Impact of impaired myocardial deformations on exercise tolerance and prognosis in patients with asymptomatic aortic stenosis. *Eur J Echocardiogr* 2009; 10:414–419. 18996958 10.1093/ejechocard/jen299

[18] Maréchaux S Carpentier E Six-Carpentier M Impact of valvuloarterial impedance on left ventricular longitudinal deformation in patients with aortic valvestenosis and preserved ejection fraction. *Arch Cardiovasc Dis* 2010; 103:227–235. 20656633 10.1016/j.acvd.2010.03.003

[19] Mizarienė V Bučytė S Zaliaduonytė-Peksienė D Components of left ventricular ejection and filling in patients with aortic regurgitation assessed by speckle-tracking echocardiography. *Medicina (Kaunas)* 2012; 48:31–38. 22481372

[20] Onishi T Kawai H Tatsumi K Preoperative systolic strain rate predicts postoperative left ventricular dysfunction in patients with chronic aorticregurgitation. *Circ Cardiovasc Imaging* 2010; 3:134–141. 20061517 10.1161/CIRCIMAGING.109.888354

[21] Baumgartner H Hung J Bermejo J Echocardiographic assessment of valve stenosis: EAE/ASE recommendations for clinical practice. *Eur J Echocardiogr* 2009; 10:1–25. 19065003 10.1093/ejechocard/jen303

[22] Zoghbi WA Enriquez-Sarano M Foster E Recommendations for evaluation of the severity of native valvular regurgitation with two-dimensional and Doppler echocardiography. *J Am Soc Echocardiogr* 2003; 16:777–802. 12835667 10.1016/S0894-7317(03)00335-3

[23] Lang RM Bierig M Devereux RB Recommendations for chamber quantification: a report from the American Society of Echocardiography's Guidelines and Standards Committee and the Chamber Quantification Writing Group, developed in conjunction with the European Association of Echocardiography, a branch of the European Society of Cardiology. *J Am Soc Echocardiogr* 2005; 18:1440–1463. 16376782 10.1016/j.echo.2005.10.005

[24] Perry GJ Helmcke F Nanda NC Evaluation of aortic insufficiency by Doppler colour flow mapping. *J Am Coll Cardiol* 1987; 9:952–959. 3558992 10.1016/s0735-1097(87)80254-1

[25] Leitman M Lysyansky P Sidenko S Two-dimensional strain—a novel software for real-time quantitative echocardiographic assessment of myocardial function. *J Am Soc Echocardiogr* 2004; 17:1021–1029. 15452466 10.1016/j.echo.2004.06.019

[26] Chen L Deng Y Liu H Speckle tracking echocardiography in evaluation of regional and global left ventricular systolic function early after lung resections. *Chinese J Intervent Imaging Ther* 2010; 7:292–295.

[27] Liu R Deng Y Liu Y Myocardial viability in patients with post-myocardial infarction using real-time myocardial contrast echocardiography and two-dimensional strain echocardiography. *Chinese J Ultrasound Med* 2009; 25:993–996.

[28] Haukoos JS Lewis RJ Advanced statistics: bootstrapping confidence intervals for statistics with “difficult” distributions. *Acad Emerg Med* 2005; 12:360–365. 15805329 10.1197/j.aem.2004.11.018

[29] Lunneborg CE *Data Analysis by Resampling: Concepts and Applications*. Pacific Grove, CA: Duxbury Press; 2000:157–166.

[30] Zhu M Ashraf M Zhang Z Real-time three dimensional echocardiographic evaluations of fetal left ventricular stroke volume, mass and myocardial strain: in vitro and in vivo experimental study. *Echocardiography* 2015; 32:1697–1706. 25865121 10.1111/echo.12939

[31] Santarpia G Scognamiglio G Di Salvo G Aortic and left ventricular remodeling in patients with bicuspid aortic valve without significant valvular dysfunction: a prospective study. *Int J Cardiol* 2012; 158:347–352. 21315467 10.1016/j.ijcard.2011.01.046

[32] Tzemos N Silversides CK Carasso S Effect of pregnancy on left ventricular motion (twist) in women with aortic stenosis. *Am J Cardiol* 2008; 101:870–873. 18328856 10.1016/j.amjcard.2007.10.054

[33] Kurt M Tanboga IH Bilen E Abnormal left ventricular mechanics in isolated bicuspid aortic valve disease may be independent of aortic distensibility: 2D strain imaging study. *J Heart Valve Dis* 2012; 21:608–614. 23167225

[34] Poulin F Carasso S Horlick EM Recovery of left ventricular mechanics after transcatheter aortic valve implantation: effects of baseline ventricular function and postprocedural aortic regurgitation. *J Am Soc Echocardiogr* 2014; 27:1133–1142. 25125314 10.1016/j.echo.2014.07.001

[35] Carasso S Cohen O Mutlak D Differential effects of afterload on left ventricular long- and short-axis function: insights from a clinical model of patients with aortic valve stenosis undergoing aortic valve replacement. *Am Heart J* 2009; 158:540–545. 19781412 10.1016/j.ahj.2009.07.008

[36] Bonow RO Picone AL McIntosh CL Survival and functional results after valve replacement for aortic regurgitation from 1976 to 1983: impact of preoperative left ventricular function. *Circulation* 1985; 72:1244–1256. 4064269 10.1161/01.cir.72.6.1244

[37] Le Tourneau T Pellikka PA Brown ML Clinical outcome of asymptomatic severe aortic stenosis with medical and surgical management: importance of STS score at diagnosis. *Ann Thorac Surg* 2010; 90:1876–1883. 21095330 10.1016/j.athoracsur.2010.07.070

[38] Yingchoncharoen T Gibby C Rodriguez LL Association of myocardial deformation with outcome in asymptomatic aortic stenosis with normal ejection fraction. *Circ Cardiovasc Imaging* 2012; 5:719–725. 23008423 10.1161/CIRCIMAGING.112.977348

